# Outcome of cell suspension allografts in a patient with Huntington's disease

**DOI:** 10.1002/ana.25354

**Published:** 2018-10-25

**Authors:** Alexander Maxan, Sarah Mason, Martine Saint‐Pierre, Emma Smith, Aileen Ho, Timothy Harrower, Colin Watts, Yen Tai, Nicola Pavese, Julie C. Savage, Marie‐Ève Tremblay, Peter Gould, Anne E. Rosser, Stephen B. Dunnett, Paola Piccini, Roger A. Barker, Francesca Cicchetti

**Affiliations:** ^1^ Centre de Recherche du CHU de Québec (CHUQ), Axe Neurosciences Québec QC Canada; ^2^ John van Geest Centre for Brain Repair and Department of Clinical Neuroscience University of Cambridge Cambridge United Kingdom; ^3^ Royal Devon and Exeter Hospital Barrack Road, Exeter Devon United Kingdom; ^4^ Department of Medicine Neurology Imaging Unit, Imperial College London London United Kingdom; ^5^ Département de médecine moléculaire Université Laval Québec QC Canada; ^6^ Laboratoire de neuropathology, Hôpital de l'Enfant‐Jésus‐CHU de Québec Québec QC United Kingdom; ^7^ Brain Repair Group and BRAIN unit, Neuroscience and Mental Health Research Institute and School of Biosciences Cardiff University Cardiff United Kingdom; ^8^ Département de Psychiatrie & Neurosciences Université Laval Québec QC Canada

## Abstract

For patients with incurable neurodegenerative disorders such as Huntington's (HD) and Parkinson's disease, cell transplantation has been explored as a potential treatment option. Here, we present the first clinicopathological study of a patient with HD in receipt of cell‐suspension striatal allografts who took part in the NEST‐UK multicenter clinical transplantation trial. Using various immunohistochemical techniques, we found a discrepancy in the survival of grafted projection neurons with respect to grafted interneurons as well as major ongoing inflammatory and immune responses to the grafted tissue with evidence of mutant huntingtin aggregates within the transplant area. Our results indicate that grafts can survive more than a decade post‐transplantation, but show compromised survival with inflammation and mutant protein being observed within the transplant site. Ann Neurol 2018;84:950–956

Huntington's disease (HD) is an autosomal‐dominant neurodegenerative disorder that presents with a combination of motor, cognitive, and psychiatric problems that progress over a 20‐year period to death. It is currently incurable, and although many therapies have been the subject of clinical trials, none have been shown to alter the natural history of this condition.^1^ More than 20 years ago, work commenced on a novel strategy that involved allografting fetal tissue into the striatum of patients with mid‐stage disease to attempt to repair the damaged circuitry, as had been shown preclinically using excitotoxic lesions of the rat and monkey striatum.^2^ To date, seven small open‐label studies of neural transplants have been conducted worldwide assessing the feasibility, safety, and tolerability of this procedure in patients with HD.^3^, ^4^, ^5^, ^6^, ^7^, ^8^, ^9^ This approach has yielded mixed results including postmortem analysis of transplanted patients. Here, we present long‐term histological data on 1 of the 5 patients treated with a fetal striatal cell suspension allograft as part of the UK study.^4^

## Materials and Methods

### *Trial Information*

This patient was 1 of 5 who were part of the NEST‐UK multicenter study, which was initiated in 1998 to evaluate the safety and efficacy of bilateral fetal striatal transplantation in patients with mild HD (ISRCTN no 36485475).^4^ The operations were undertaken in Cambridge between 2000 and 2003, and this report deals with patient number 5, the only one to receive bilateral transplants of fetal striatal tissue in a single operation. The trial was approved by the Cambridge Regional Ethics Committee (REC ref: 95/086), as was the postmortem analysis (REC ref:01/117).

### *Tissue Preparation and Neurosurgical Procedures*

Details of tissue procurement and preparation, immunosuppression, safety assessment, and implantation have been fully reported elsewhere.^4^, ^10^, ^11^, ^12^ The patient was followed up using the standard CAPIT‐HD protocol.^4^, ^5^

### *Postmortem Histological Evaluation*

The brain was removed 37.3 hours after death and processed as described in a previous work.^13^ Sections were initially prepared for histochemical analyses to assess graft location, survival, and cyto‐architecture. For this, one section of each graft was stained for acetylcholinesterase (AChE), as described in a previous publication.^16^

### *Immunohistochemistry*

For immunohistochemical stainings, the following primary antibodies were used: the neuronal marker microtubule‐associated protein 2 (MAP2; rabbit anti‐MAP2; 17490‐1‐AP, 1:500; Proteintech, Chicago, IL) or neuronal nuclei (NeuN; mouse anti‐NeuN; MAB377, 1:2500; Millipore, Burlington, MA) with the mutant huntingtin (mHtt) antibody (EM48; MAB5374, 1:500; Millipore, Burlington, MA). For striatal projection neurons, we used the calcium‐binding protein calbindin (rabbit anti‐CB; Ab11426, 1:1,000; Abcam, Cambridge, MA) and the dopamine‐ and cAMP‐regulated neuronal phosphoprotein (DARPP‐32; rabbit anti‐DARPP‐32; 2306, 1:1,000; Cell Signaling Technology, Danvers, MA). To identify grafted interneurons, we used calretinin (rabbit anti‐CR; 7699/4, 1:1,000; Swant, Marly, Switzerland), parvalbumin (mouse anti‐PV; P3088, 1:1,000; Sigma‐Aldrich, St. Louis, MO), and choline acetyltransferase (mouse‐anti‐ChAT; MAB5270, 1:200; Millipore), as previously described.^14^ We also performed a histochemical staining for nicotinamide adenine dinucleotide phosphate (NADPH‐d)—a marker for nitric oxide containing striatal interneurons—following previously published protocols.^14^, ^15^ The Inflammatory/immune response was visualized using the microglial marker, ionized calcium‐binding adaptor molecule 1 (Iba1; rabbit anti‐Iba1; LAK4357, 1:500; Wako Chemicals USA, Richmond, VA), the T‐helper cell marker, CD4 (mouse anti‐CD4; NCL‐L‐CD4‐368, 1:500; Leica Microsystems, Wetzlar, Germany), and the natural killer and cytotoxic T cells, CD8 (mouse anti‐CD8; NCL‐L‐CD8‐4B11, 1:500; Leica Microsystems). Sections stained for immune cells (CD4 and CD8) were further pretreated in a 10‐mM sodium citrate buffer solution at 80°C for 20 minutes. In some cases, immune cells were also counterstained with Nissl. Finally, a single immunohistochemistry staining for tyrosine hydroxylase (TH; rabbit anti‐TH; P40101‐150, 1:1,000; Pel Freeze Biologicals, Rogers, AR) was used to assess the dopaminergic innervation of the graft. In all cases, sections were incubated with appropriate secondary antibodies (biotinylated goat antimouse immunoglobulin G [IgG]; BA9200 1:500; Vector Laboratories, Burlingame, CA; biotinylated goat antirabbit IgG; BA1000 1:500; Vector Laboratories) using the ABC Elite Vectastain Kit (Vector Laboratories).

### *Image Acquisition and Quantification*

Brightfield photomicrographs were taken using the Picture Frame software (MicroBrightField Bioscience, Williston, VT) attached to an E800 Nikon microscope (Nikon Instruments, Tokyo, Japan) and prepared for illustration using Adobe Photoshop CS5 and final figure panels assembled using Adobe Illustrator CS5 (Adobe Systems, San Jose, CA).

All cell quantifications were performed on two sections of the cortex and striatum containing the graft. The perimeters of the grafted areas were delineated using the tracing contour option in Stereo Investigator (NeuroExplorer, version 10.0; MicroBrightField Bioscience) as described in a previous publication.^16^ To measure cholinergic activity, images of AChE stainings were processed with ImageJ software (NIH, Bethesda, MD) and P‐zones, non‐P‐zones (NP‐zones), and the host striatum were individually traced. These areas were measured for their mean gray values of color intensity.

### *Statistical Analysis*

For all cell quantifications, an unpaired Student *t* test was performed using Prism (6.0; GraphPad Software Inc., La Jolla, CA).

## Results

### *Clinical Course*

The patient first noticed problems in 1995, at the age of 37, with a slight change in his mood and his family became aware of his problems in 1997 when he developed nightmares and depression. He had a family history for HD and went on to have a positive genetic test with an expansion of 47 CAG repeats in exon 1 of the huntingtin gene. In 2003, he was selected for neural grafting and underwent a bilateral transplant procedure without complications and was followed up according to the CAPIT‐HD protocol until his death in 2015. His clinical history showed no obvious change in his disease course after grafting, either clinically or on positron emission tomography imaging, as detailed in a previous work.^4^

### *Postmortem Graft Evaluation*

#### *Graft Location and Cytoarchitecture*

**Figure 1:**
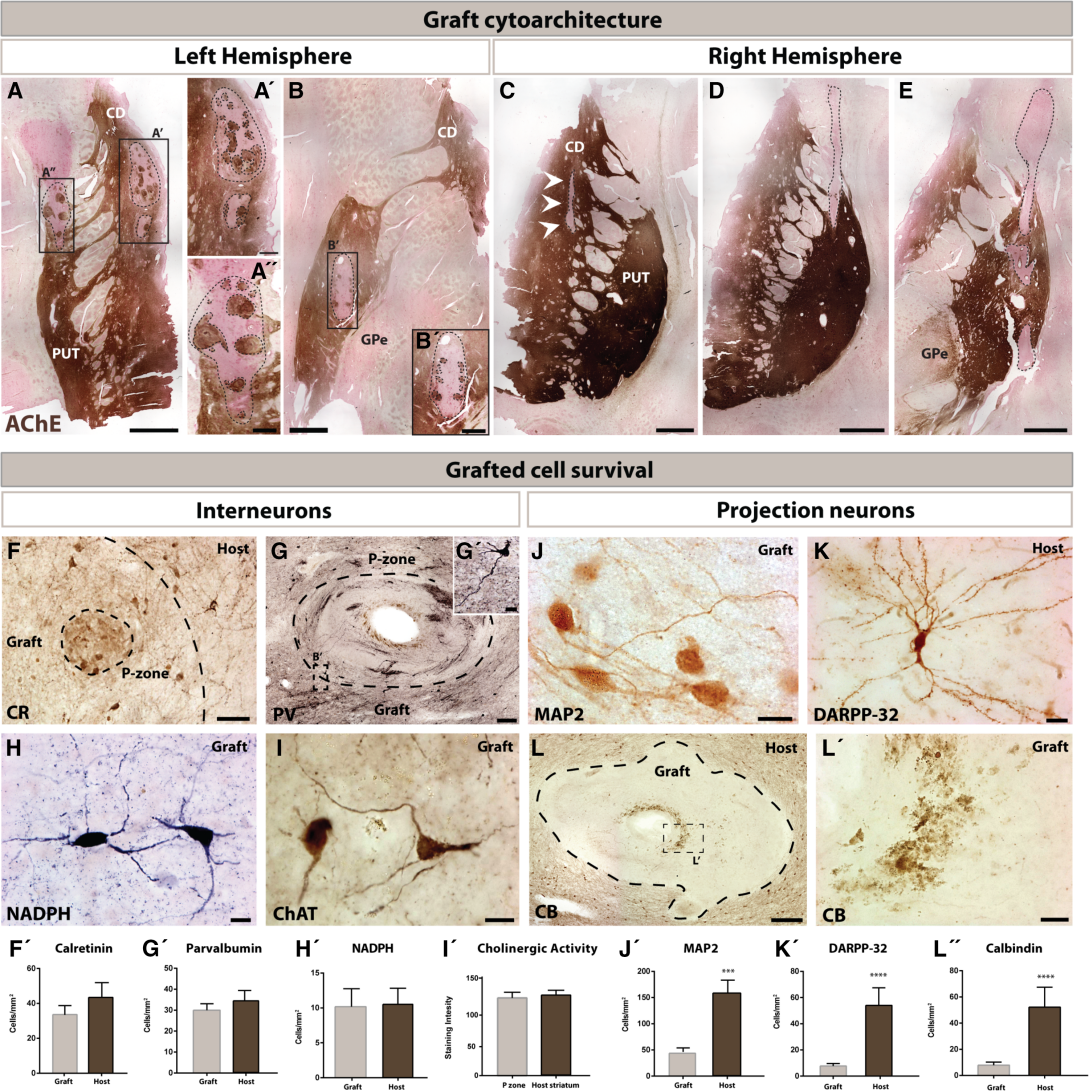
Graft location, cytoarchitecture and grafted cell survival. Macroscopic identification of the transplants based on AChE staining revealed a total of three grafts in the left hemisphere (delineated by dotted lines): one in the upper caudate nucleus (A,A′) and two in the putamen (A,A″,B,B′) that occupied between 7.2% and 9.9% of the total striatal area. Cell suspension grafts were observable as clusters closely resembling P‐zones and NP‐zones (A,B′). An additional three grafts were located in the right hemisphere (delineated by dotted lines): one in the caudate nucleus (C) and two in the putamen (D,E) that were much smaller and occupied less than 1.2% of the total striatal area. Immunohistochemical staining for striatal interneurons included CR, PV, NADPH, and ChAT (F–I). High‐power photomicrographs highlighting the CR (F) and PV (G) staining of cells within a p‐zone of the graft as well as examples of grafted neurons expressing NADPH‐d (H), or ChAT (I). All grafted interneurons showed a rather healthy morphology with extensive dendritic arborizations. Stereological cell counts revealed a similar number of CR‐ (F′), PV‐ (G′), NADPH‐ (H′), and ChAT‐immunolabeled (I′) cells in the grafted area vs the host striatum. Similar immunohistochemical staining approaches were used to identify grafted projection neurons and included MAP2, DARPP‐32, and CB (K–L′). Whereas certain P‐zones displayed a restricted number of healthy MAP2 staining cells (J), DARPP‐32^+^ projection neurons were typically absent in the grafted tissue, but frequently found within the host striatum (K). In contrast to interneurons, detectable CB‐immunoreactive projection neurons were largely necrotic (L,L′). Stereological cell counts revealed a striking difference between the number of MAP2‐ (J′), DARPP‐32‐ (K′), and CB‐immunolabeled (L″) elements in the grafted area vs the host striatum. Scale bars: A,B,C,D,E = 1.25mm; A′,B′ = 250 µm; A″ = 20 µm; F = 100 µm; G = 250 µm; H,I = 25 µm; J = 25 µm; K = 50 µm; L = 250 µm; L′ = 50 µm. Abbreviations: AChE = acetylcholinesterase; CB = calbindin; CD = caudate nucleus; ChAT = choline acetyltransferase; CR = calretinin; DARPP‐32 = dopamine‐ and cAMP‐regulated neuronal phosphoprotein; GPi = globus pallidus internal segment; GPe = globus pallidus external segment; MAP2 = microtubule‐associated protein 2; NADPH = nicotinamide adenine dinucleotide phosphate; PV = parvalbumin; PUT = putamen.

Macroscopically grafts were easily identified. In total, six grafts were located in the left hemisphere with two in the caudate and four in the putamen (Fig 1A,B′) whereas in the right hemisphere only one and two grafts were found in these structures respectively (Fig 1C–E).

#### *General Graft Health*

All the typical phenotypes of striatal interneurons were observed within the grafted tissue including cells positive for calretinin (CR), parvalbumin (PV), NADPH, and ChAT (Fig 1F–I). They showed healthy morphologies and extensive dendritic arborizations. Stereological quantifications for each of these cell types revealed similar counts within the transplanted area in comparison to the host striatum for CR, PV, and NADPH interneurons (Fig 1F′–I′). Despite rare examples of healthy MAP2 staining within the graft (Fig 1J), the presence of DARPP‐32–positive cells was almost exclusively observed within the host striatum (Fig 1K). Grafted projections neurons, as also identified with calbindin (CB), were largely necrotic (Fig 1L,L′) and there were fewer cells within the transplant area. Because of the tested antibody being discontinued, we used AChE staining to measure cholinergic enzyme levels within the P‐zones and compared them to the host striatum, where they looked similar (Fig 1I′).

#### *Inflammatory‐Immune Response to Cell Suspension Grafts*

**Figure 2:**
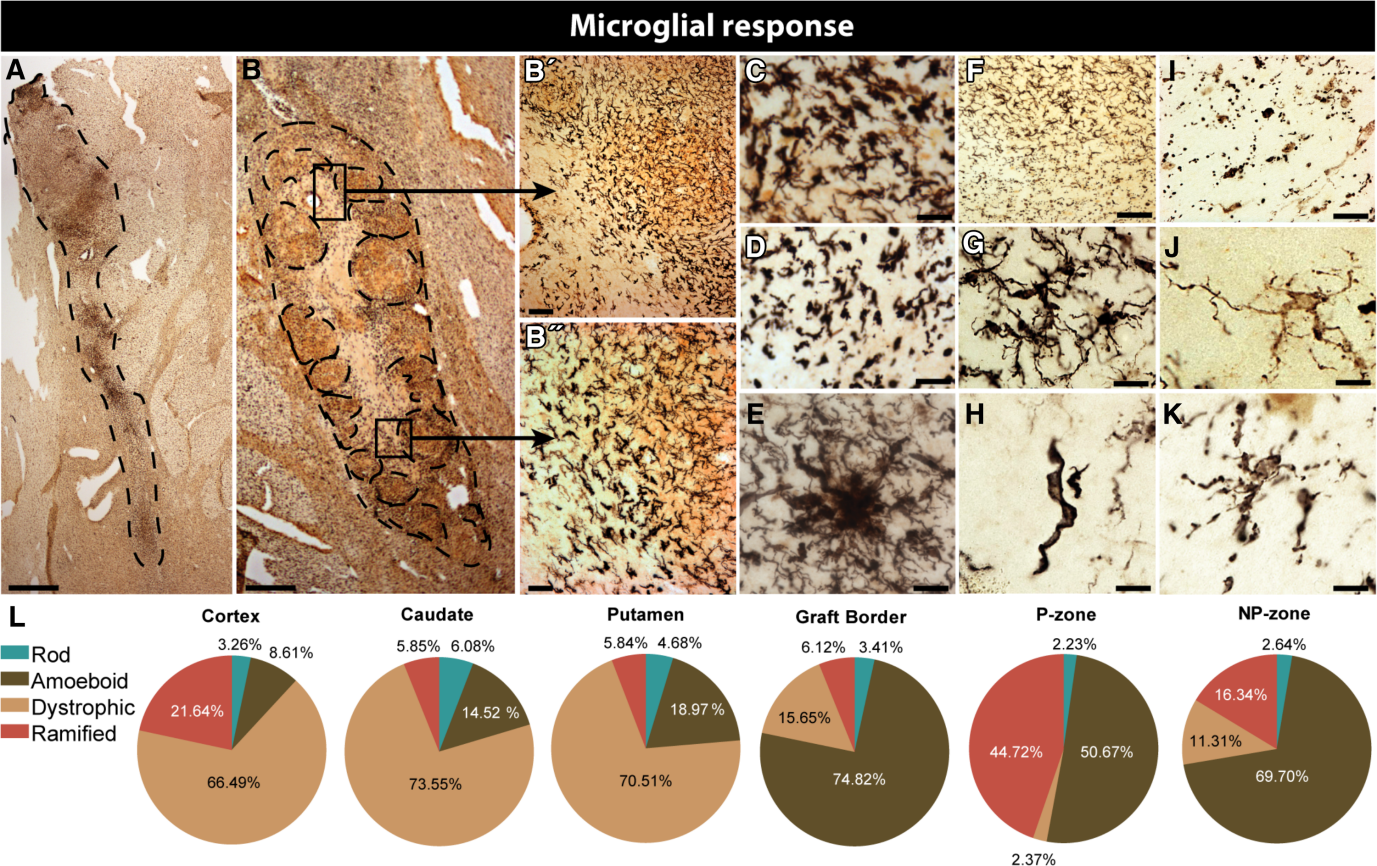
Inflammatory response to cell suspension grafts. Series of double immunostainings to visualize inflammatory response to grafted cells. (A,B) Double immunohistochemical staining for Iba1 using nickel‐intensified DAB (microglial cells in black) and NeuN as revealed using chromogen DAB (neuronal cell in brown) in selected grafts. (B,B′) Representative images of the clear gradient in microglial response intensity (C) as well as various microglial morphologies within and surrounding the grafts. (C–K) Higher‐power magnification of grafted areas, showing microglial activation at the graft‐host border (C,E), within P‐zones (D), NP‐zones (F), clusters of activated cells (G), and host cells (H), illustrating the intensity of the microglial response as well as the various phenotypes of the activated cells. Proinflammatory microglia are identifiable by the increased Iba1 reactivity and shortened process length (E). Highly dystrophic microglia in the host were often missing processes entirely (H) or otherwise displaying beaded, broken ramifications (I). Slightly activated microglia (J) and rod cells (K) were present within NP‐zones. (L) Pie charts detailing the percentages of microglial cell populations found at different structures or graft interfaces. Scale bars: A,B = 1mm; B′ = 50 µm; B″ = 35 µm; C,D,E,I = 30 µm; F = 80 µm; G = 20 µm; H,J,K = 15 µm. DAB = 3,3′‐diaminobenzidine; Iba1 = ionized calcium‐binding adaptor molecule 1; NeuN = neuronal nuclei.

A ring of densely packed, maximally hyperactive microglia surrounded each of the transplanted sites (Fig 2A,B) and was also present within the graft itself. Throughout this ring, the spectrum of microglial activation was represented from dystrophic (Fig 2C,D), clustered (Fig 2E) and rod (Fig 2H) cells. Grafted regions lacking striatal markers (NP‐zones) had qualitatively much lower microglial density, though microglial morphology and Iba1 intensity remained consistent with a proinflammatory phenotype (Fig 2F,G). As senescence continues, these processes become fragmented and severe dystrophy was confirmed by spherical microglial cell bodies with no processes, surrounded by small dots of cytoplasm apparently disconnected from the soma (Fig 2I). The outer borders of the graft contained more dystrophic microglia, with gnarled, beaded processes (Fig 2J,K). The host tissue, on the other hand, was almost entirely populated by dystrophic and nonfunctional microglia. The P‐zones contained the highest ratio of ramified microglia and the fewest dystrophic cells (Fig 2L), although there were still many more hyperactive cells than normally present in healthy tissue.

### *Graft Innervation and Presence of HD‐Related Pathology*

**Figure 3:**
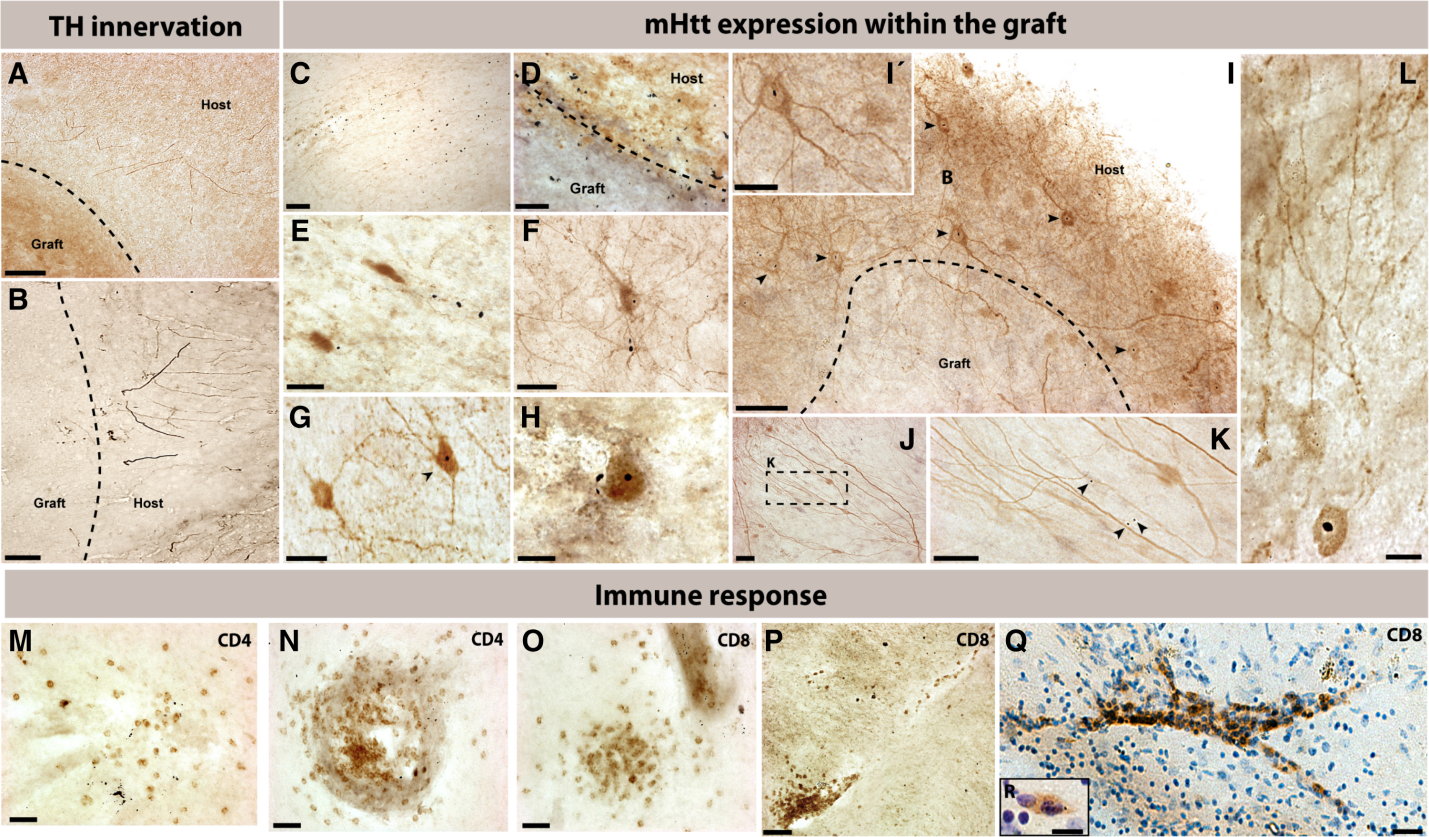
Graft innervation, presence of HD‐related pathology, and T‐cell response to cell suspension grafts. (A,B) Tyrosine hydroxylase immunostaining demonstrated modest innervation of the grafts by the dopaminergic system of the host. In some grafts (C), as well as at the border of the grafts (D), the presence of mHtt was abundant. Within the grafted area, mHtt was observable both within the extracellular matrix (E,F) and also within the nucleus of a number of cells within the graft site (F–H, chevrons). In addition to being localized within cell bodies of the host (I,I′), mHtt was observable within (J,K) and along dendritic paths of cells within the graft or within cells that had a complex arborization (L). Both CD4 (M,N) and CD8 staining (O–R) revealed clusters of infiltrating cells within the grafts and around blood vessels found within the transplants. On rare occasions, mHtt was observed within CD8^+^ cells (R). Scale bars: A = 175 µm; B,C = 100 µm; D,I,J,K = 50 µm; E,F,I′,L = 25 µm; G = 35 µm; H = 20 µm. M,N,O = 60 µm; P = 100 µm; Q = 30 µm; R = 8 µm. Abbreviations: CD4 = cluster of differentiation 4; CD8 = cluster of differentiation 8; mHtt = mutant huntingtin protein; TH = tyrosine hydroxylase.

The grafts received modest innervation, as evidenced by the sparse dopaminergic fibers observed near the transplants (Fig 3A,B). Several mHtt^+^ aggregates were observed within the graft site and at the graft/host interface (Fig 3C,D). The mHtt^+^ aggregates were not only observed within the extracellular matrix of the grafted area, as previously reported in solid tissue transplants,^16^ but they were also found within cell bodies in the grafts (Fig 3F–H,L). A particularly interesting observation was that some mHtt^+^ aggregates were found along, or even within, the dendritic tree of cells within the transplant (Fig 3J,K). Notably, striatal host cells expressing mHtt^+^ extended their dendritic trees into the grafts (Fig 3I,I′).

Finally, groups of T‐helper cells (stained for cluster of differentiation 4 [CD4]; Fig 3M,N) and cytotoxic T cells (stained for cluster of differentiation 8 [CD8]; Fig 3O–R) were found forming clusters within the grafts, indicating an ongoing immune and inflammatory response at the time of death. Of significance, mHtt, was also identified within infiltrating T‐helper cells (Fig 3R).

## Discussion

This is the first histological report on striatal cell suspension allografts originating from the UK NEST striatal transplantation HD project (NEST‐HD).^5^, ^17^ Postmortem evaluation revealed that (1) it was easy to find striatal transplants within which grafted interneurons were largely spared in contrast to the grafted projection neurons that were rarely observed; (2) there was a striking microglial response around the graft; (3) there were mHtt aggregates within the grafted tissue as well as within cells of the transplanted area and finally; and (4) there was infiltration of immune cells within the grafted tissue that also contained mHtt, both of which may have further contributed to poor graft survival. These observations are similar to those that we have reported previously in HD patients who receiving solid tissue pieces, but, importantly, there were notable differences in that we saw a much more marked infiltration of immune cells in the cell suspension grafts along with more florid mHtt cellular pathology within the transplant sites.

This latter observation relating to mHtt expression within the grafts requires further comment. One could argue that the cells expressing mHtt within the boundaries of the grafted areas are host cells that have migrated into the transplant. Alternatively, one could speculate that innervation of the grafts by host striatal neurons containing mHtt^+^ results in the transport of the pathological protein from the host to the graft, given that mature striatal cells have little if any migratory capacity.^18^ Unfortunately, the underlying explanation for this finding cannot be explored in this context, because the technical limitations inherent to human postmortem analysis do not allow one to make mechanistic conclusions. Additionally, other routes of transport should be considered in light of the fact that mHtt^+^ was observed, although rarely, within CD8^+^ cells. The significant infiltration of CD4^+^ and CD8^+^ cells, notable within the grafted areas, may have indeed been facilitated by the more complex and efficient vascularization that we observed within the cell suspension grafts (data not shown).

In conclusion, this study again highlights that fetal striatal allografts can survive long term in the human HD brain. However, although interneurons within them survive, projection neurons degenerate, and this is all associated with inflammation around and in the transplant as well as the expression of mHtt pathology at the graft site. The relevance and mechanistic consequences of these observations awaits clarification, but raises questions as to whether cell‐based approaches for repairing the HD brain can ultimately repair the dysfunctional networks observed in this condition.^16^, ^19^, ^20^, ^21^, ^22^, ^23^, ^24^, ^25^, ^26^

## Author Contributions

S.L.M. and R.A.B. were involved in the study concept and design. A.M., M.S.‐P., E.S., A.H., T.H., C.W., Y.T., N.P., P.P., J.S., M.‐E.T., and P.G. were involved in data acquisition and analysis. A.M., S.L.M., R.A.B., and F.C. were involved in drafting of the manuscript and figures.

## Potential Conflicts of Interest

Nothing to report.

## References

[1] Mason SL , Barker RA . Advancing pharmacotherapy for treating Huntington's disease: a review of the existing literature. Expert Opin Pharmacother 2016;17:41–52. 26536068 10.1517/14656566.2016.1109630

[2] Kendall AL , Rayment FD , Torres EM , Baker HF , Ridley RM , Dunnett SB . Functional integration of striatal allografts in a primate model of Huntington's disease. Nat Med 1998;4:727–729. 9623985 10.1038/nm0698-727

[3] Bachoud‐Levi A , Bourdet C , Brugieres P , et al. Safety and tolerability assessment of intrastriatal neural allografts in five patients with Huntington's disease. Exp Neurol 2000;161:194–202. 10683285 10.1006/exnr.1999.7239

[4] Rosser AE , Barker RA , Harrower T , et al.; NEST‐UK. Unilateral transplantation of human primary fetal tissue in four patients with Huntington's disease: NEST‐UK safety report ISRCTN no 36485475. J Neurol Neurosurg Psychiatry 2002;73:678–685. 12438470 10.1136/jnnp.73.6.678 PMC1757375

[5] Barker RA , Mason SL , Harrower TP , et al. The long‐term safety and efficacy of bilateral transplantation of human fetal striatal tissue in patients with mild to moderate Huntington's disease. J Neurol Neurosurg Psychiatry 2013;84:657–665. 23345280 10.1136/jnnp-2012-302441 PMC3646287

[6] Kopyov OV , Jacques S , Lieberman A , Duma CM , Eagle KS . Safety of intrastriatal neurotransplantation for Huntington's disease patients. Exp Neurol 1998;149:97–108. 9454619 10.1006/exnr.1997.6685

[7] Hauser RA , Sandberg PR , Freeman TB , Stoessl AJ . Bilateral human fetal striatal transplantation in Huntington's disease. Neurology 2002;58:1704; author reply, 1704. 10.1212/wnl.58.11.1704 12058114

[8] Gallina P , Paganini M , Lombardini L , et al. Human striatal neuroblasts develop and build a striatal‐like structure into the brain of Huntington's disease patients after transplantation. Exp Neurol 2010;222:30–41. 20026043 10.1016/j.expneurol.2009.12.005

[9] Capetian P , Knoth R , Maciaczyk J , et al. Histological findings on fetal striatal grafts in a Huntington's disease patient early after transplantation. Neuroscience 2009;160:661–675. 19254752 10.1016/j.neuroscience.2009.02.035

[10] Rosser AE , Barker RA , Armstrong RJ , et al. Staging and preparation of human fetal striatal tissue for neural transplantation in Huntington's disease. Cell Transplant 2003;12:679–686. 14653615 10.3727/000000003108747299

[11] Farrington M , Wreghitt TG , Lever AM , Dunnett SB , Rosser AE , Barker RA . Neural transplantation in Huntington's disease: the NEST‐UK donor tissue microbiological screening program and review of the literature. Cell Transplant 2006;15:279–294. 16898222 10.3727/000000006783981927

[12] Watts C , Donovan T , Gillard JH , et al. Evaluation of an MRI‐based protocol for cell implantation in four patients with Huntington's disease. Cell Transplant 2003;12:697–704. 14653617 10.3727/000000003108747316

[13] Cisbani G , Freeman TB , Soulet D , et al. Striatal allografts in patients with Huntington's disease: impact of diminished astrocytes and vascularization on graft viability. Brain 2013;136(pt 2):433–443. 23378216 10.1093/brain/aws359

[14] Cicchetti F , Saporta S , Hauser RA , et al. Neural transplants in patients with Huntington's disease undergo disease‐like neuronal degeneration. Proc Natl Acad Sci U S A 2009;106:12483–12488. 19620721 10.1073/pnas.0904239106 PMC2713393

[15] Braak H , Alafuzoff I , Arzberger T , Kretzschmar H , Del Tredici K . Staging of Alzheimer disease‐associated neurofibrillary pathology using paraffin sections and immunocytochemistry. Acta Neuropathol 2006;112:389–404. 16906426 10.1007/s00401-006-0127-z PMC3906709

[16] Cicchetti F , Lacroix S , Cisbani G , et al. Mutant huntingtin is present in neuronal grafts in Huntington disease patients. Ann Neurol 2014;76:31–42. 24798518 10.1002/ana.24174

[17] HDCRB . A novel gene containing a trinucleotide repeat that is expanded and unstable on Huntington's disease chromosomes. The Huntington's Disease Collaborative Research Group. Cell 1993;72:971–983. 8458085 10.1016/0092-8674(93)90585-e

[18] Li JY , Englund E , Holton JL , et al. Lewy bodies in grafted neurons in subjects with Parkinson's disease suggest host‐to‐graft disease propagation. Nat Med 2008;14:501–503. 18391963 10.1038/nm1746

[19] Desplats P , Lee HJ , Bae EJ , et al. Inclusion formation and neuronal cell death through neuron‐to‐neuron transmission of alpha‐synuclein. Proc Natl Acad Sci U S A 2009;106:13010–13015. 19651612 10.1073/pnas.0903691106 PMC2722313

[20] Hansen C , Angot E , Bergstrom AL , et al. alpha‐Synuclein propagates from mouse brain to grafted dopaminergic neurons and seeds aggregation in cultured human cells. J Clin Invest 2011;121:715–725. 21245577 10.1172/JCI43366 PMC3026723

[21] de Calignon A , Polydoro M , Suarez‐Calvet M , et al. Propagation of tau pathology in a model of early Alzheimer's disease. Neuron 2012;73:685–697. 22365544 10.1016/j.neuron.2011.11.033 PMC3292759

[22] Luk KC , Kehm V , Carroll J , et al. Pathological alpha‐synuclein transmission initiates Parkinson‐like neurodegeneration in nontransgenic mice. Science 2012;338:949–953. 23161999 10.1126/science.1227157 PMC3552321

[23] Luk KC , Kehm VM , Zhang B , O'Brien P , Trojanowski JQ , Lee VM . Intracerebral inoculation of pathological alpha‐synuclein initiates a rapidly progressive neurodegenerative alpha‐synucleinopathy in mice. J Exp Med 2012;209:975–986. 22508839 10.1084/jem.20112457 PMC3348112

[24] Meyer‐Luehmann M , Coomaraswamy J , Bolmont T , et al. Exogenous induction of cerebral beta‐amyloidogenesis is governed by agent and host. Science 2006;313:1781–1784. 16990547 10.1126/science.1131864

[25] Meyer‐Luehmann M , Stalder M , Herzig MC , et al. Extracellular amyloid formation and associated pathology in neural grafts. Nat Neurosci 2003;6:370–377. 12598899 10.1038/nn1022

[26] Jeon I , Cicchetti F , Cisbani G , et al. Human‐to‐mouse prion‐like propagation of mutant huntingtin protein. Acta Neuropathol 2016;132:577–592. 27221146 10.1007/s00401-016-1582-9 PMC5023734

